# A bilateral neoplasm in chest: a case report and literature review

**DOI:** 10.1186/1471-2482-14-42

**Published:** 2014-07-09

**Authors:** Cheng Shen, Zhaojie Han, Guowei Che

**Affiliations:** 1 Department of Thoracic Surgery, West-China Hospital, Sichuan University, Chengdu 610041, China

**Keywords:** Myelolipoma, Pulmonary, Mediastinum

## Abstract

**Background:**

Myelolipoma is a rare neoplasm composed of yellowish adipose tissue and reddish-brown tissue corresponding to hematopoietic or hemorrhages. It typically occurs in adrenal glands as a solitary, well-circumscribed mass, and the thoracic location is extremely unusual.

**Case presentation:**

We present a rare case who is a 54 years old male with bilateral Myelolipoma of the posterior mediastinum. He underwent the surgery via video-assisted thoracic surgery both sides interval 3 months. Histological examination showed both tumors consisted of mature fat tissue and hematopoietic tissue, including myeloid, erythroid, and megakaryocytic elements surrounded. We discussed the etiology, histopathology, differential diagnosis and recommended management of extra-adrenal myelolipoma and analyzed the features of the thoracic myelolipoma including mediastinal and pulmonary location.

**Conclusions:**

Literature review showed 16 similar cases, with a 2/1 male/female ratio and a mean age of 58 years. Eight of sixteen cases were observed in the mediastinum and six of sixteen cases were displayed in the pulmonary and one showed on the chest wall. CT and MRI scans are able to indicate the presence of extra-adrenal myelolipoma. Pathological analysis is an effective method to clarify the diagnosis. Observation and surgery are two regular treatment methods. Small, asymptomatic tumors should be monitored, while large tumors that cause unendurable symptoms may be removed by surgery.

## Background

Myelolipoma is encapsulated and composed of yellowish adipose tissue and reddish-brown tissue corresponding to hematopoietic or hemorrhages [1]. It was first reported by Gierke in 1905 and its generally accepted name, “myelolipoma” was purposed by Oberling in 1929 [2]. Most myelolipomas were confronted incidentally during autopsy and their incidence was estimated to vary from 0.01% to 0.2%. The most common site of involvement is the adrenal gland. Extra-adrenal myelolipomas may occur in the retroperitoneum, pelvis, presacral area, stomach, liver and even in the thyroid glands; they are seldom seen in the thorax [3]. Herein we report on a rare case of a bilateral myelolipoma, extra-adrenal myelolipoma (EAM) located in the posterior mediastinum. And we also try to analyze the features of the thoracic EAMs include mediastinal and pulmonary location.

## Case presentation

**Figure 1:**
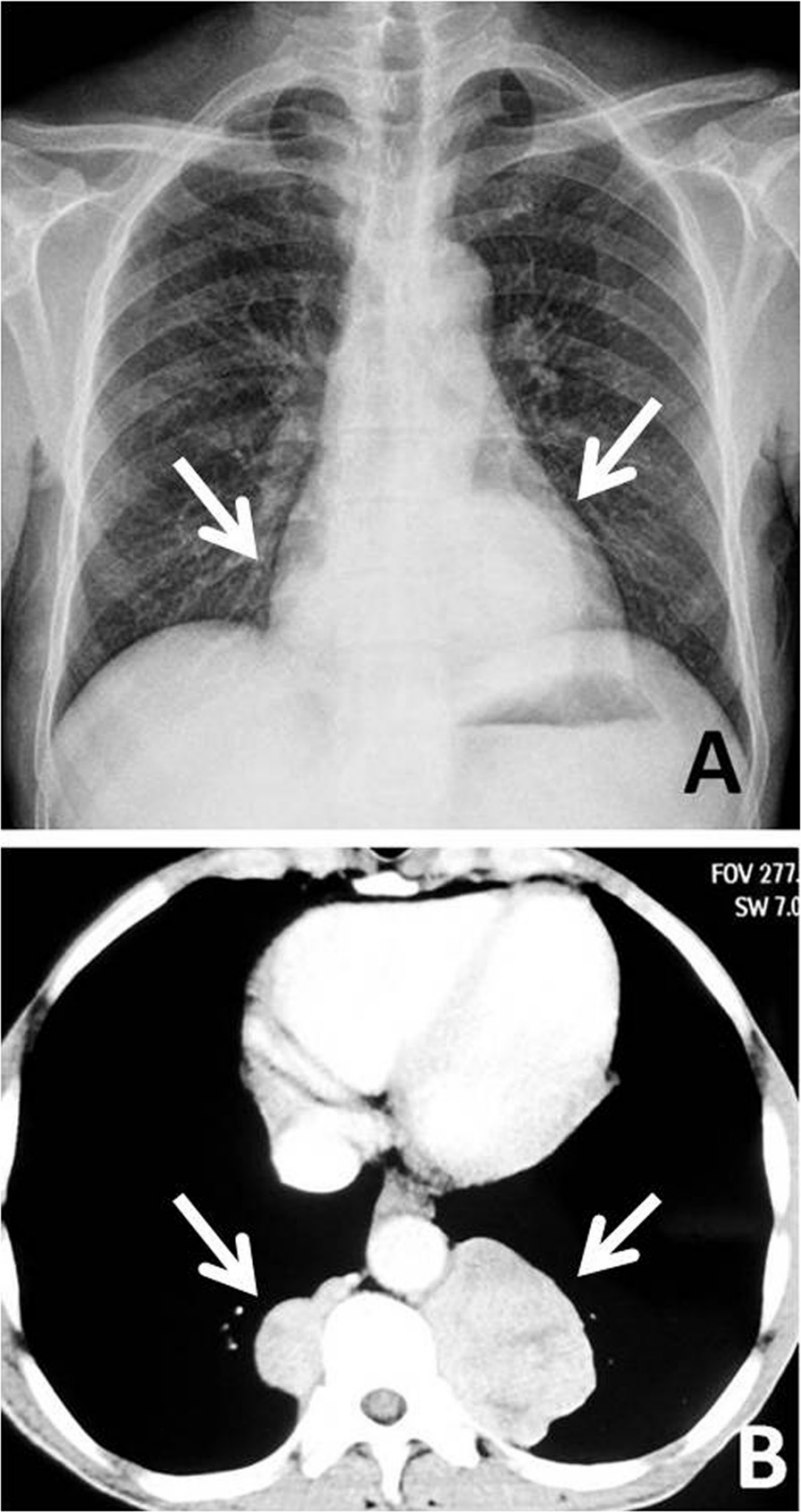
**Chest X-ray and contrast-enhanced CT features of the case. A**: Posteroanterior chest X-ray showing a bilateral mass in the posterior mediastinum. **B**: Contrast-enhanced CT scan showing a bilateral thoracic paravertebral mass.

**Figure 2:**
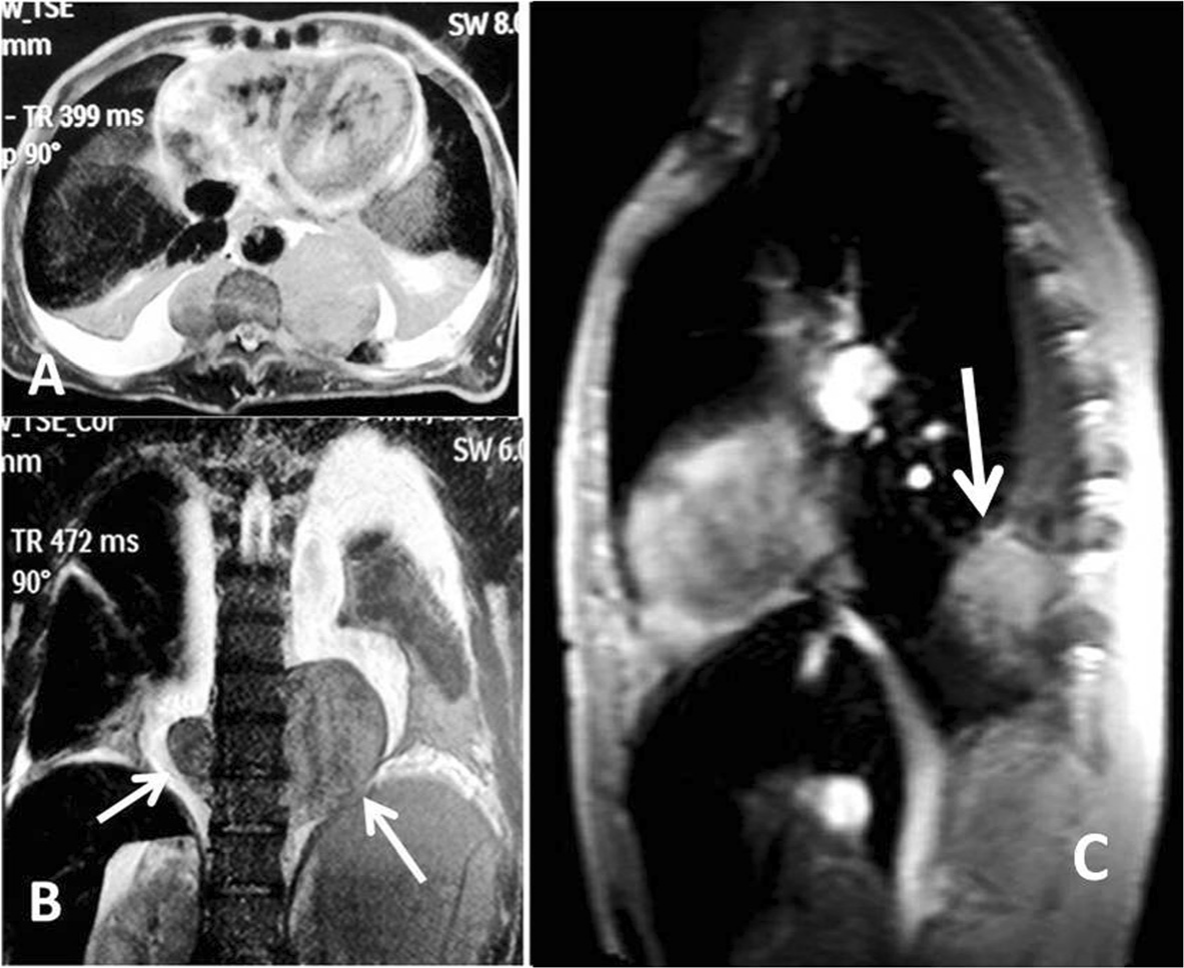
**MRI features of the case. A**: MRI scans reveals two masses in the posterior mediastinum. **B**: MPR on coronal plane shows a bilateral thoracic paravertebral mass. **C**: MPR on sagittal plane confirms the left mass in the posterior mediastinum.

**Figure 3:**
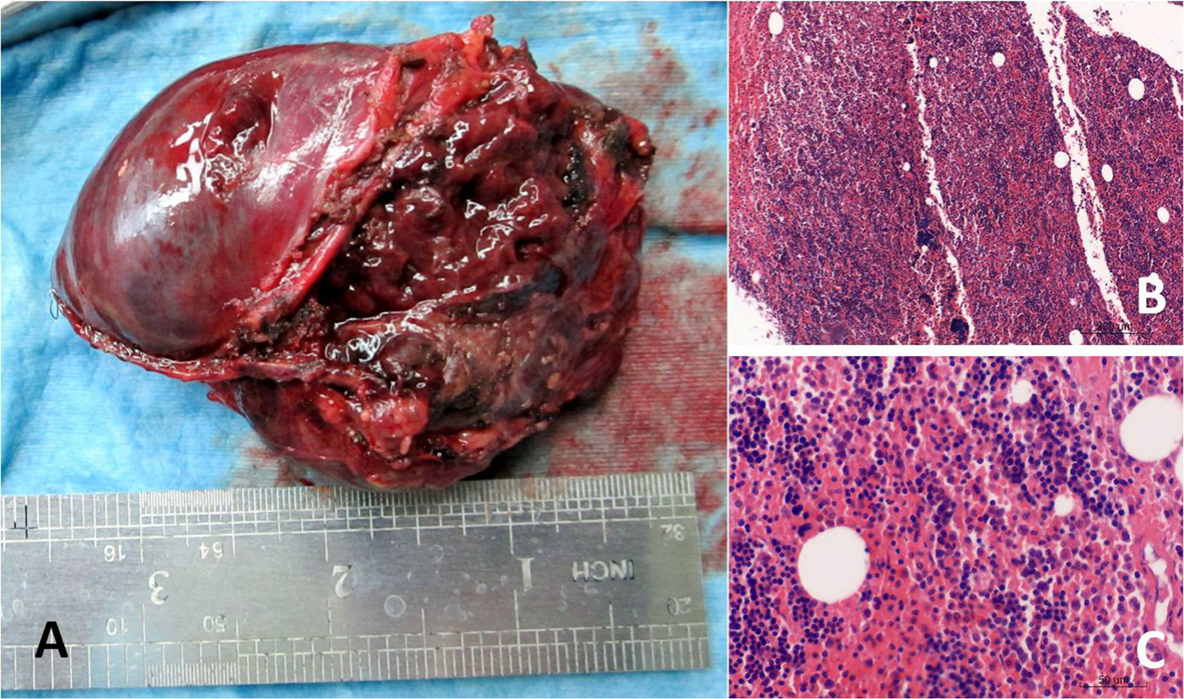
**Histological and immunoistochemical features. A**: The freshly resected mass showing its circumscription. **B**: H&E staining reveals hematopoietic elements admixed with adipose tissue (X100). **C**: High power magnification (X400) shows very few adipocytes admixed with hemotopoietic elements, including megakaryocytes and cells of regular granulopoietic and erythropoietic cell lineages.

A 54-year-old man was referred to our hospital for assessment of two posterior mediastinal masses that was detected on chest radiography during a routine health check. He denied the following symptoms including the presence of chest pain, hoarseness, hemoptysis, cough and dyspnea. He was a non-smoker and had no exposure to any environmental fumes or dust. Physical examination revealed normal breath sounds in both of the lung fields. Laboratory findings were within normal limits. In these examinations, hematology tests and biochemistry tests were within regular levels. His Pulmonary function tests and cardiovascular examination revealed normal performance. Plain chest computed tomography (CT) scans two paravertebral masses with well-defined margins, well demarcated from the descending aorta in the posterior mediastinum measuring 7.0 × 5.5 cm of the left one and 3.8 × 2.8 cm of the right one in size. There was no destruction of adjacent bone structures, no pleural effusion or infiltration of surrounding tissues and no evidence of intraforaminal or intraspinal tumor manifestation. Contrast-enhanced CT scans showed obvious enhancement in soft tissue masses (Figure 1A, 1B). Magnetic resonance imaging (MRI) included axial scan, and multiplanar reconstructions (MPRs) on the coronal and sagittal planes (Figure 2A, 2B, 2C) revealed that both of the masses extending from levels T9-10 in the coronal planes. Mixed-signal lesions, mainly equal to a T1/T2 signal, were observed. In the marginal regions, a cystic lesion exhibited longer T1 and T2. There was no involvement of the vertebral canal and the bony structure was normal.As diagnosis was not established through imaging, surgery was scheduled. We approached the left tumor which was removed by en bloc resection via video-assisted thoracic surgery (VATS) rather than the thoracotomy. There was no invasion into the adjacent structures. After careful attention to hemostasis, a chest tube was placed to drain the pleural cavity, and the left lung was reinflated under direct vision. Three months later the right-sided lesion of 3.8 cm in diameter was also excised. Grossly, both tumors disclosed a thin capsule of soft consistency and reddish-brown color. Microscopically, both tumors consisted of mature fat tissue and hematopoietic tissue, including myeloid, erythroid, and megakaryocytic elements surrounded (Figure 3A, 3B, 3C). Based on these findings, extra-adrenal myelolipoma was diagnosed. The postoperative course was uneventful. The patient was followed up for 5 months without evidence of recurrence to date.

## Conclusions

Myelolipoma is a relatively uncommon benign tumor, composed of mature adipose tissue and hematopoietic elements. Myelolipoma occurs at any age, which varied from 12 to 93 years [2,4], however, patients with EAM are generally older (mean age 64 years) than patients with adrenal myelolipoma (mean age 55 years) [5]. The male to female ratio ranged from 2:1 to 1:1 [5,6]. As in our analysis, the male to female ratio was 2:1 and the mean age was 58 years (Table 1).

**Table 1:** Clinicopathological features of the primary myelolipomas in thorax

**Case**	**Age (years)**	**Sex**	**Location**	**Number**	**Tumor size (cm)**	**Treatment**	**Follow up**	**Author**
**1**	68	M	Right posterior mediastinum	Single	Not determined	VATS	Alive	Ema T [7]
**2**	56	M	Posterior mediastinum	Single	25*15	Thoracotomy	Alive	Vaziri M [8]
**3**	65	F	Bilateral of mediastinum	Mutiple	φ4.5/φ6.5	Thoracotomy	Alive	Franiel T [9]
**4**	55	M	Posterior mediastinum	Single	φ4	VATS	Alive	Koizumi J [10]
**5**	53	F	Pleura	Single	Not determined	Thoracotomy	Alive	Spanta R [11]
**6**	50	M	Bilateral of mediastinum	Mutiple	Not determined	Thoracotomy	Alive	Krismann M [12]
**7**	71	F	Right anterior mediasinum	Single	6*5.5*5.5	Thoracotomy	Dead	Krag D [13]
**8**	68	F	Bilateral of mediastinum	Multiple	3.1*10/2.5*9	Thoracotomy	Alive	Geng C [14]
**9**	46	M	Right anterior mediastinum	Single	φ4.5	Thoracotomy	Alive	Mohan K [15]
**10**	57	F	Right low lobe	Single	13*10*2.5	VATS	Alive	Huang W [16]
**11**	53	M	Left upper lobe	Single	2.3*1.2*1.0	Thoracotomy	Alive	Huang WT [17]
**12**	63	M	Chest wall	Single	2.0*1.5*1.2	VATS	Alive	Sagan D [18]
**13**	70	M	Left low lobe	Single	2	Chemotherapy	Dead	Sato K [19]
**14**	45	M	Left upper lobe	Single	φ1.5	Thoracotomy	Alive	Lu X [20]
**15**	54	M	Pulmonary	Single	Not determined	Thoracotomy	Alive	Sabate CJ [21]
**16**	52	M	Pulmonary	Single	Not determined	Thoracotomy	Dead	Zunarelli E [22]

The exact pathogenesis of myelolipoma is not clear at present. Historically, there are 3 theories of the pathogenesis of myelolipomas. The first is that myelolipomas are derived from bone marrow emboli that lodge in the adrenal gland or other sites. The second theory suggests that myelolipomas are derived from embryonic primitive mesenchymal cells. The third theory suggests that myelolipomas arise from metaplastic transformation of adrenal (or other sites) stromal cells in response to stimuli. As Dieckmann KP *et al.*[23] reported, the hypotheses of the cause of EAM include degenerative changes in hyperplastic tumor cells, metaplasia in primary stem mesenchymal cells of the adrenal cortex, and displacement of differentiated bone marrow cells during embryogenesis. Chen and colleagues suggested that in patients with anemia, long-lasting erythropoietic stimulation may play a role in the development of EAM [24]. Once in a while, EAM may be accompanied by endocrine disorders like Cushing’s syndrome, Addison’s disease, Cohn’s syndrome, phaeochromocytoma, diabetes mellitus, or even obesity and hypertension [25]. Therefore, some authors emphasize that development of EAM may be correlated with prolonged excessive steroid production, or genome defects of the endocrine glands responsible for multiple endocrine neoplasia type 1 [24].

EAMs are typically asymptomatic. They tend to be incidentally discovered during radiological investigation of symptoms unrelated to myelolipoma. In our analysis, most of the patients are asymptomatic [7,8,10-12,18,21,22]. But some patients with mediastinum myelolipoma presented with productive cough [9], the stiff neck [13], dull back pain and a cough [14] or central chest pain [15]. The patients with pulmonary myelolipoma complain of fever [16], productive cough [17,20] or intractable lumbago [19] and the others were asymptomatic (Table 1). There was no pain or cough in our case caused by the lesion, but it was detected from the chest radiography during a routine health check.

Because these tumors are rare, criteria for diagnosing EAM radiologically do not exist. Characteristics of myelolipoma are deduced to apply to these structures which present in a variety of locations [26]. Chest radiographs are the most generally performed imaging study to evaluate the mass, but it may not be possible to distinguish myelolipoma from others. Myelolipoma can be reliably diagnosed by either computed Tomography (CT) or magnetic resonance imaging (MRI). Radiological imaging typically displays a well-circumscribed mass with a heterogeneous appearance due to the varying proportions of fat within the mass. Adipose tissue is characterized by low attenuation on CT imaging (i.e., -25 to -100 Hounsfield units). On MRI, fat displays high signal intensity on T1-weighted images whereas the myeloid component of these tumors has a T2-weighted signal. Contrast enhancement with CT scan or MRI will vary depending on the composition of the mass. Soft tissue components enhance whereas adipose tissue does not [27]. There is also a word of caution in our case that patients with myelolipoma are not only examined with axial scan but also checked with MPRs on coronal plane and sagittal plane, because they can be useful in preoperative diagnosis.

There is no agreement on how to manage EAM. Daneshmand *et al.*[28] suggested that small asymptomatic tumors (<4 cm in size) should be monitored, while symptomatic tumors or large myelolipomas (>7 cm in size) should be removed. Considering the potential progressive enlargement of the lesion and the uncertain preoperative diagnosis, most myelolipomas are surgically removed, which is a useful method for treating myelolipomas and the tumors generally do not recur, and in recent years this operation has frequently been performed by using Video Assisted Thoracic Surgery (VATS). In our table, only four patients were operated by Video Assisted Thoracic Surgery VATS and the others were treated with thoracotomy. According to the report of Sato K [19], the 70-year-old patient presented with intractable lumbago and MRI detected multiple bone tumours. CT of the chest indicated a large mass in the right lower lobe and a small solitary nodule with rim-like calcification in the left low lobe of the lung. Sputum cytology showed carcinoma cells. He was clinically diagnosed as having lung cancer with multiple bone metastases. Chemotherapy and radiation therapy had no effect, and the patient died of respiratory failure. An autopsy was performed after death and the small solitary nodule was diagnosed as myelolipoma. In the present case, the male whose CT and MRI scans revealed two posterior mediastinal masses was operated by VATS. A roundish, paravertebral, encapsulated solid mass of soft consistency measuring 7.0 cm in diameter was removed. Three months later the right-sided lesion of 3.8 cm in diameter was also excised.

Grossly, EAM is a solitary circumscribed mass ranging in size from a few centimeters to 27 cm [29]. From our table, the size of the masses ranged from 1 cm to 25 cm. The tumor is usually spherical to ovoid, well circumscribed, sometimes surrounded by a pseudocapsule. The cut surface typically has a variegated appearance, with areas of greasy-appearing soft yellow tissue alternating with irregular areas of dark red-brown friable tissue. Microscopically, the tumor is composed of a variable admixture of mature adipose tissue with islands and nests of hematopoietic elements of different percentages. The cellularity of hematopoietic precursors is variable and the three hematopoietic cell lineages (granulopoietic, erythropoietic and megakaryocytic) are present. In some cases, areas of infarction, hemorrhage, and rarely foci of calcification are noted [30]. Bony spicules were rarely noted and were only found in large tumors [18].

When the diagnosis of myelolipoma is considered, it should be differentiated from other tumors. Pulmonary myelolipoma should be distinguished from extramedullary hematopoiesis and hamartoma. Extramedullary hematopoiesis usually occurs as a manifestation of myeloproliferative diseases or is a compensatory phenomenon in various chronic anemias. In contrast to myelolipoma, extramedullary hematopoiesis of the lung frequently presents with multiple occurrences and ill-defined lesions. Microscopically, extramedullary hematopoiesis is composed predominantly of hematopoietic cells and erythroid hyperplasia. Lung hamartomas generally consist of cartilage, fat, and connective tissue [31]. When myelolipoma is composed predominantly of fat tissue, it is difficult to distinguish it from Liposarcoma. Macroscopically, a liposarcoma consists usually of a large, well-circumscribed, lobulated mass. Color varies from yellow to white depending on the proportion of adipocytic, fibrous and/or myxoid areas. Differential diagnosis of subpleural chest wall masses should include lesions of similar clinical appearance, such as lipomas, lymph node metastases, and in particular, neurogenic neoplasms. Posterior mediastinal EAM must be considered in preoperative differential diagnosis, to differentiate it, not only from extramedullary hematopoietic tumors but also from other tumors occurring at these sites such as lymph node metastases, lymphomas or neurogenic tumors.

The usual natural history of EAM is benign although they may enlarge and bleed. Some reports have shown stable lesions with follow up from 3 to 62 months [18]. Small asymptomatic lesions may be managed expectantly. Radiological follow up is recommended due to the potential for growth and hemorrhage. As in ours, surgical intervention is warranted to the patients who have an unclear diagnosis or possess an enlarging tumor mass. Long-term prognosis is excellent.

Although primary extra-adrenal myelolipoma is rare, understanding its diagnosis and treatment are important. If a patient is suspected to have primary extra-adrenal myelolipoma by CT or MRI scans, this may be clarified it by pathological analysis. Subsequently, the tumor may be modified or removed by surgery, according to its effects on the body.

### Consent

Written informed consent was obtained from the patient for publication of this case report and any accompanying images. A copy of the written consent is available for review by the Editor-in-Chief of this journal.

## Competing interests

The authors declare that they have no competing interests.

## Authors’ contributions

CS was involved in drafting the manuscript. ZH was involved in acquisition of data and preparing the figures. GC designed and revised the manuscript. All authors have read and approved the final manuscript.

## Pre-publication history

The pre-publication history for this paper can be accessed here:

http://www.biomedcentral.com/1471-2482/14/42/prepub
